# LGD-DeepLabV3+: An Enhanced Framework for Remote Sensing Semantic Segmentation via Multi-Level Feature Fusion and Global Modeling

**DOI:** 10.3390/s26031008

**Published:** 2026-02-03

**Authors:** Xin Wang, Xu Liu, Adnan Mahmood, Yaxin Yang, Xipeng Li

**Affiliations:** 1 College of Information and Technology, Jilin Agricultural University, Changchun 130118, China; liuxu@mails.jlau.edu.cn (X.L.); 20231314@mails.jlau.edu.cn (Y.Y.); 20241269@mails.jlau.edu.cn (X.L.); 2 School of Computing, Macquarie University, Sydney, NSW 2109, Australia; adnan.mahmood@mq.edu.au

**Keywords:** remote sensing, semantic segmentation, DeepLabV3+, multi-level feature fusion, global context modeling

## Abstract

Remote sensing semantic segmentation encounters several challenges, including scale variation, the coexistence of class similarity and intra-class diversity, difficulties in modeling long-range dependencies, and shadow occlusions. Slender structures and complex boundaries present particular segmentation difficulties, especially in high-resolution imagery acquired by satellite and aerial cameras, UAV-borne optical sensors, and other imaging payloads. These sensing systems deliver large-area coverage with fine ground sampling distance, which magnifies domain shifts between different sensors and acquisition conditions. This work builds upon DeepLabV3+ and proposes complementary improvements at three stages: input, context, and decoder fusion. First, to mitigate the interference of complex and heterogeneous data distributions on network optimization, a feature-mapping network is introduced to project raw images into a simpler distribution before they are fed into the segmentation backbone. This approach facilitates training and enhances feature separability. Second, although the Atrous Spatial Pyramid Pooling (ASPP) aggregates multi-scale context, it remains insufficient for modeling long-range dependencies. Therefore, a routing-style global modeling module is incorporated after ASPP to strengthen global relation modeling and ensure cross-region semantic consistency. Third, considering that the fusion between shallow details and deep semantics in the decoder is limited and prone to boundary blurring, a fusion module is designed to facilitate deep interaction and joint learning through cross-layer feature alignment and coupling. The proposed model improves the mean Intersection over Union (mIoU) by 8.83% on the LoveDA dataset and by 6.72% on the ISPRS Potsdam dataset compared to the baseline. Qualitative results further demonstrate clearer boundaries and more stable region annotations, while the proposed modules are plug-and-play and easy to integrate into camera-based remote sensing pipelines and other imaging-sensor systems, providing a practical accuracy–efficiency trade-off.

## 1. Introduction

High-resolution remote sensing semantic segmentation plays a pivotal role in numerous real-world applications, including urban planning, land-use monitoring, disaster response, agricultural management, and environmental protection [1]. Advanced imaging sensors—such as spaceborne optical cameras, airborne push broom scanners, and UAV-borne digital payloads—now provide large-area coverage with fine ground sampling distances, often reaching centimeters. However, achieving accurate pixel-level semantic understanding remains challenging due to significant scale variations, high intra-class diversity coupled with inter-class similarity, severe shadow occlusions caused by terrain or buildings, and pronounced domain shifts from heterogeneous sensor characteristics, including varying viewing geometries, spectral responses, and point-spread functions. In complex scenes, slender linear structures and intricate man-made boundaries are prone to fragmentation or blurring, while dense urban areas coexisting with sparse rural regions often lead to severe class imbalance. Seasonal illumination changes and atmospheric effects further degrade local contrast and edge fidelity, demanding models that preserve fine-grained local details and enforce long-range semantic consistency across vast spatial extents.

Recent work in sensors has shown that even carefully designed networks still struggle to balance multi-scale feature modeling and boundary sharpness in high-resolution land-cover scenes [2,3,4]. To tackle these challenges, research has explored diverse enhancement strategies across multiple stages of the segmentation pipeline. In terms of backbone architecture innovation, Wang et al. propose UNetFormer, an efficient UNet-style transformer that streamlines the decoder and sustains real-time segmentation on urban imagery [5]. Liu et al. design AMTNet with attention-based multiscale transformers in a CNN and Transformer Siamese framework to enhance multi-level context aggregation [6]. Liu et al. develop CSWin-UNet, integrating cross-shaped-window attention into a U-shaped architecture to better capture elongated patterns under controlled complexity [7]. Fan et al. introduce DDPM-SegFormer, which fuses diffusion features with a transformer to enhance land-use and land-cover delineation [8]; Chen et al. propose CTFuse, a serial CNN–Transformer hybrid with spatial and channel attention that attains competitive results on ISPRS Potsdam and Vaihingen [9].

On the input side, researchers increasingly address photometric degradation and distribution shift prior to feature extraction. He et al. propose a lightweight restoration method tailored to remote sensing imagery using axial depthwise convolutions coupled with hybrid attention to improve visibility under haze and occlusions at modest cost [10]. Ma et al. develop a decomposition-based unsupervised domain adaptation approach for remote sensing image semantic segmentation [11]. Xu et al. leverage Fourier-based augmentation to align amplitude statistics and correct distribution mismatches, improving cross-domain robustness [12]. In parallel, dynamic and sparse strategies, such as dynamic token pruning, prune or select tokens to contain the cost of global modeling while retaining long-range dependencies [13]. In addition to single-modal enhancements, multimodal fusion networks have demonstrated improved robustness to shadows and sensor-specific artifacts, as illustrated by MMFNet [14].

For the decoder and boundary-refinement stage, in-network mechanisms continue to offer improvements in thin-structure continuity and edge fidelity. Xiao et al. introduce BASeg, which employs a boundary-refined module combined with context aggregation to enhance object boundaries and fine structures in complex scenes [15]. Li et al. propose an edge-aware multi-task learning method that jointly predicts masks, edges, and distance maps to regularize boundaries and close small parcels [16]. Qu et al. present FBRNet, which incorporates a border-refinement module with feature-fusion and multi-scale context aggregation to preserve fine structures and sharp boundaries in challenging scenarios [17].

Despite these advances, existing methods exhibit critical limitations for practical deployment in remote sensing workflows. Transformer-based and Mamba-based architectures excel in global context modeling through long-range dependencies, yet they often incur substantial computational complexity and memory footprint, which may require nontrivial adaptations for very large remote sensing tiles and established engineering pipelines. Input preprocessing and decoder refinement techniques, although effective in isolation, frequently deliver only marginal overall gains and fail to address cross-stage interactions comprehensively. Moreover, many recent works sacrifice inference efficiency in pursuit of sophisticated modules, making them less suitable for latency-sensitive or resource-constrained applications that rely on the efficient and widely adopted CNN + ASPP paradigm.

To address these shortcomings while retaining the merits of the classic DeepLabV3+ framework—namely its computational efficiency, modular design, and seamless integration into existing remote sensing processing pipelines—this paper proposes LGD-DeepLabV3+, an enhanced semantic segmentation model with three lightweight, pluggable modules targeting complementary stages. The Laplacian-guided Input Shaping (LIS) module employs multi-level Laplacian pyramid decomposition followed by nonlinear remapping via KAN convolutions to reshape the raw input distribution, suppressing cross-frequency interference and enhancing early feature separability without altering the backbone interface. This stabilizes optimization for heterogeneous sensor data and preserves high-frequency details crucial for boundary delineation. The Global Context Routing (GCR) module, inserted after the Atrous Spatial Pyramid Pooling (ASPP), adopts a demand-driven two-stage routing strategy to model near-global dependencies at low cost, ensuring cross-region semantic consistency while avoiding quadratic complexity of full self-attention. The Directional Perception Fusion (DPF) module refines shallow-deep feature fusion in the decoder through coordinate-aware directional recalibration, local refinement, and cross-layer channel coupling, prioritizing elongated structures and true boundaries while suppressing texture-induced noise. All three modules are sensor-agnostic, are designed as plug-and-play components, and maintain full compatibility with the original DeepLabV3+ tensor interfaces, facilitating integration into diverse remote sensing systems. Although this work is developed on the efficient CNN + ASPP pipeline, the proposed modules are formulated as plug-and-play components, and their interfaces do not inherently restrict them to CNN backbones. Extensive experiments on representative remote sensing benchmarks validate the effectiveness and generality of the proposed framework. Detailed experimental settings, baselines, and quantitative results are reported in the Experiments section.

The remainder of this paper is organized as follows: Section 2 elaborates on the proposed methodology; Section 3 presents experimental setups, comparisons, qualitative results, and ablation analyses; and Section 4 concludes with discussions on limitations and future directions.

## 2. Methods

### 2.1. Overall Framework of LGD-DeeplabV3+

**Figure 1:**
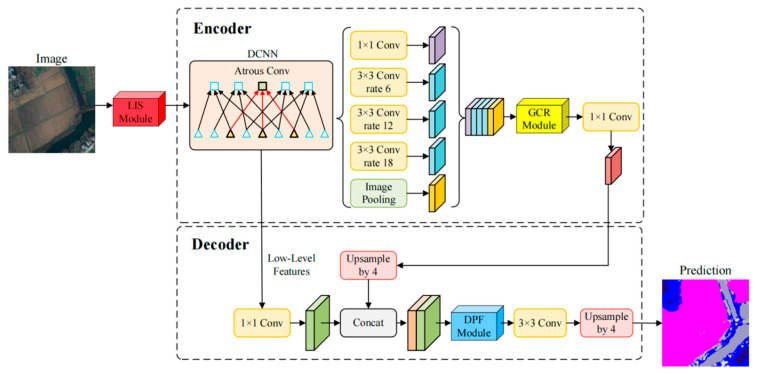
General framework of LGD-DeepLabV3+ with three improved instrumentation locations. Arrows indicate the feature flow direction, and dashed boxes denote the encoder and decoder stages. Squares and triangles in the DCNN illustrate different sampling positions in atrous convolution.

This paper takes the encoder–decoder paradigm of DeepLabV3+ as the baseline [18]. The input image is first processed by the convolutional backbone to extract features, and the ASPP at the top layer performs multi-scale context aggregation. The decoder retrieves details from earlier layers and concatenates them with the upsampled semantic layers, ultimately producing pixel-level predictions. Without changing the main structure or tensor interfaces, we insert lightweight and pluggable enhancement modules at three key locations, forming LGD-DeepLabV3+, as shown in Figure 1. Because all three modules are implemented as generic convolution and attention operators that act on image tensors, they are agnostic to the underlying imaging sensor and can, in principle, be applied to RGB/IRRG aerial cameras, multispectral satellite sensors, and multimodal image–DSM pairs without modifying the backbone interface. These three locations are the input side before the backbone, the context stage after ASPP, and after the shallow-deep feature concatenation in the decoder.

The input side is handled by the LISModule, which performs feature re-shaping using Laplacian-pyramid decomposition to obtain high-frequency details and low-frequency residuals, preserving fine structures and global context. This method has been effective in segmentation tasks [19]. These components are then reshaped nonlinearly and compressed into channels using lightweight KAN convolutions while maintaining the spatial resolution. The features at each scale are upsampled to a consistent size and later fused laterally to produce reshaped features of the same size as the input. This process, implemented before entering the backbone, achieves weak decoupling and distribution reshaping, allowing high-frequency textures to be preserved without excessive magnification, while low-frequency structures are calibrated to a more regular statistical state. As a result, it provides a more optimized starting point for subsequent feature learning and maintains consistency with the input channels and data format of the backbone.

After the ASPP, the GCRModule is introduced to reinforce long-range dependencies and global consistency. This module adopts a two-stage routing strategy that first performs coarse selection and then fine-grained computation. In the coarse-grained stage, the feature map from ASPP is divided into a regular grid, and candidate region scores are generated for each query position. Only the top-scoring candidates are retained to form the attention range. The fine-grained stage then executes multi-head attention and linear transformations within the union of these candidate regions, completing information exchange. This is followed by residual connections and normalization to stabilize training, and the output is mapped back to the required number of channels in the backbone using a 1 × 1 convolution. The demand-driven routing ensures that each position connects only with a few relevant areas, maintaining near-global perception while keeping computational and memory costs within acceptable limits [20]. As the working resolution is consistent with ASPP, the multi-scale features from GCRModule and ASPP complement each other without requiring changes to the backbone or tensor sizes.

In the decoding stage, the DPFModule performs direction-sensitive recalibration, local refinement, and cross-layer channel interactions. After shallow details and deep semantics are concatenated at the same resolution, spatial recalibration is performed through coordinate decomposition, followed by 1D global pooling along the row and column directions to obtain two context clues sensitive to direction. These shallow and deep branches form four attention branches—shallow row, shallow column, deep row, and deep column—to generate fine-grained spatial guidance maps, performing direction-sensitive recalibration on the concatenated features. Building on this, two layers of 3 × 3 convolutions are stacked, or layer-wise depth-wise convolutions are used to achieve lightweight processing, emphasizing real boundaries while suppressing false responses caused by textures. Shallow and deep channels are then globally pooled separately to form compact channel representations, which are linearly mapped to obtain mutually gated channel weights, allowing shallow details and deep semantics to align cooperatively along the channel dimension. The DPFModule prioritizes maintaining boundaries and slender structures, significantly improving the stability of shallow-deep fusion with minimal increases in parameters and computational cost. The module’s output is then directly connected to the 3 × 3 convolution and upsampling path of DeepLabV3+, maintaining interface consistency.

The three modules together form LGD-DeepLabV3+. The input reshaping stabilizes the data distribution and reduces optimization difficulty, while the routing-based global modeling in the context stage captures cross-region relationships more efficiently. The direction-sensitive fusion in the decoder strengthens the delineation of real boundaries and small targets. The entire solution preserves the end-to-end training flow and is easy to integrate and migrate into existing systems while being more computationally and memory-efficient [21]. We next detail each component and the training objectives.

### 2.2. LISModule

**Figure 2:**
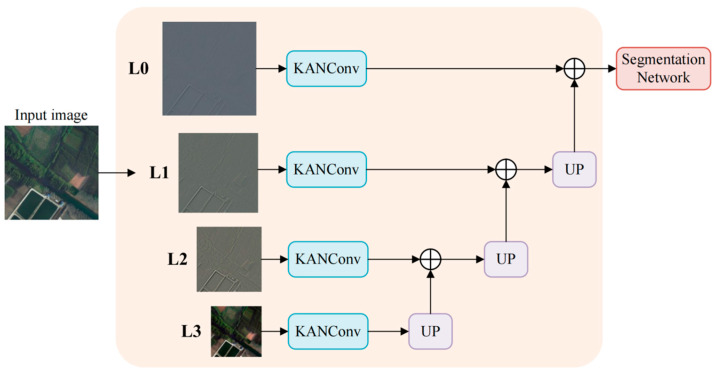
The overall process of LIS. The input image undergoes a four-level Laplacian decomposition to obtain *L*0, *L*1, *L*2, and *L*3. KANConv is applied within each scale to complete nonlinear reshaping and channel compression. The features are then progressively upsampled to the original resolution and fused laterally. The output maintains consistency in both size and channels with the input interface of the backbone.

To reduce optimization difficulty before entering the backbone and ensure both large-area consistency and boundary distinguishability, the input side adopts a pre-processing strategy of four-level Laplacian decomposition followed by layer-wise KAN convolutions. First, the image is decomposed into multi-frequency components using a four-level Laplacian pyramid [22]. Then, multi-scale information is fused at the original resolution. Finally, KAN convolution is applied for lightweight shaping and channel alignment, and the resulting features are passed to the DeepLabV3+ backbone. This is the overall process of the Laplacian-guided Input Shaping (LIS) Module, as shown in Figure 2.

The input image $x\in R^{H\times W\times 3}$ is first processed by a four-level Laplacian pyramid to complete frequency decomposition and separation. The input image is first decomposed into multiple frequency bands by a four-level Laplacian pyramid. Let $G_{l}$ represent the l-th high-pass filter in the pyramid. The filter used has a kernel of *h* = [1, 4, 6, 4, 1]/16, with a 5 × 5 size, and is calculated by:

(1) $$G_{0}=x,G_{l+1}=Down2(G_{l}*h),l=0,1,2$$

The corresponding Laplacian components are defined by:

(2) $$L_{l}=G_{l}-Up2(G_{l+1}*h),l=0,1,2,L_{3}=G_{3}$$

For each component at each scale, a KAN convolution operator, denoted as KANConv [23], is introduced. It approximates nonlinear mapping and performs channel compression using learnable basis functions within a fixed local neighborhood. The goal of LIS is not to extract high-level semantics, but to re-map multi-scale Laplacian residuals into a more stable and separable distribution before the backbone. At this stage, the desired operation is closer to frequency-dependent normalization and contrast recalibration than to spatial pattern matching. Compared with standard convolutions that mainly rely on fixed linear filtering followed by pointwise activation, KANConv performs a learnable basis-function remapping, enabling flexible yet smooth nonlinear transformations of residual amplitudes. This helps suppress extreme responses caused by illumination/sensor differences while preserving informative high-frequency structures. Therefore, the KAN-based remapping can be viewed as a frequency-aware amplitude normalization on Laplacian residuals, which reduces cross-frequency interference and aligns residual statistics across domains. Let the receptive field size *k* be 3, and the number of basis functions *M* be 6. Then, for any position *p* and output channel *o*, the output is given by:

(3) $$y_{o}(p)=\sum_{c=1}^{C_{l}}\underset{u\in Nk}{\sum }\sum {m=1}^{M}a_{o,c,u,m}B_{m}(L_{l,c}\;(p+u))+b_{o}$$

Here, Nk represents the k × k neighborhood, and $\left\{B_{m}(\cdot )\right\}$ are the fixed B-spline bases, with a and b being learnable coefficients. Let the output of this scale be denoted as $F^{l}=KANConv(L_{l})$, and then $\left\{F^{l}\right\}$ are progressively upsampled to the original resolution and later fused horizontally to form intermediate features. Let $F^{↑l}$ denote the feature obtained by upsampling $F^{l}\;$ ell from scale l to the reference resolution. This is given by:

(4) $$F^{↑l}={Up2}^{\left(l\right)}(F^{l}),F=\phi (\sum_{l=0}^{3}F^{↑l})$$

The term ${Up2}^{\left(l\right)}$ represents upsampling to the same resolution as the input by a factor of $2^{l}$, where ϕ denotes the use of Batch Normalization (BN) and GELU. To match the main interface, the final 1 × 1 convolution maps the channels to the expected input channels while keeping the spatial size H×W unchanged.

### 2.3. GCRModule

**Figure 3:**
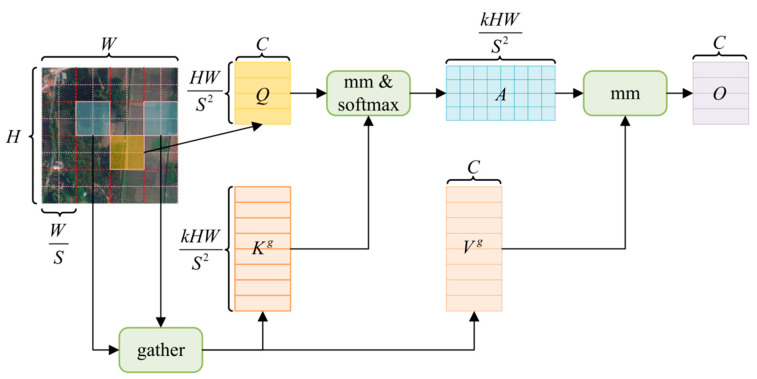
The overall process of the two-stage routing attention in the GCRModule. Let the output resolution of ASPP be H × W (i.e., 1/16 scale). The features are divided into an S × S grid of regions, and *Top-k* routing is performed based on region similarity. Attention is calculated only on the union of the candidates and then projected back to the channel dimension. Colored boxes highlight example regions, and the red dashed lines indicate the partition of the feature map into an S × S grid.

To further model long-range dependencies while maintaining low computational overhead after ASPP, this paper inserts a Global Context Routing (GCR) Module at the 1/16 scale. The core idea is to first perform coarse filtering followed by fine-grained calculations. Initially, candidate routing is selected at the region level, and then fine-grained attention is applied only to the union of these candidates. This allows each position to interact with only a small number of relevant regions, thereby approximating global perception while significantly reducing irrelevant computations [24]. The process is shown in Figure 3.

Let the output of ASPP be ${\;X}_{aspp}\in R^{H\times W\times C}$ (where H,W are the height and width of the original image, respectively, and the scale is 1/16). The feature map is divided into a non-overlapping grid of S×S, and the region index $R=\frac{H}{S}\cdot \frac{W}{S}$. The key, value, and other computations are derived from:

(5) $$Q=XW_{q},\;\;\;\;\;K=XW_{k},\;\;\;\;\;V=XW_{v},{\;\;\;\;\;W}_{q},W_{k},W_{v}\in R^{C\times C}$$

Each region-level grid is averaged to obtain the region-level representation $Q_{r}$, $K_{r}\in R^{R\times C}$, where R is the grid size and C is the number of channels. The region similarity $A_{r}=Q_{r}K_{r}^{⊤}$ is computed, and for each query region, the *top-*k most relevant regions are selected from a row of $A_{r}$, obtaining the selection set $I_{r}=TopK(A_{r},k)$. Then, the detailed computation on the selected set is performed, where the corresponding keys and values are collected based on $I_{r}$, forming the compact candidate union $K_{g}{,V}_{g}$. The attention points are summed up and projected back into the original channel. Meanwhile, 3 × 3 depthwise convolutions are applied to enhance spatial locality, suppress noise, and strengthen the boundaries. The model uses Pre-Norm residuals and trains with a two-layer MLP, as described in:

(6) $$X_{1}=X_{0}+{DWConv}_{3\times 3}(LN(X_{0}))$$

The routing attention interaction is given by:

(7) $$U1=LN(X_{0}),Q,K,V=Linear(U1)$$

(8) $$O=Softmax(\frac{QK_{g}^{⊤}}{\sqrt{d}})V_{g}+{DWConv}_{3\times 3}(V)$$

(9) $$X_{2}=X_{1}+O$$

The channel reconstruction output is given by:

(10) $$X_{gcr}=X_{2}+MLP(LN(X_{2})),X_{gcr}\in R^{H\times W\times C}$$

**Figure 4:**
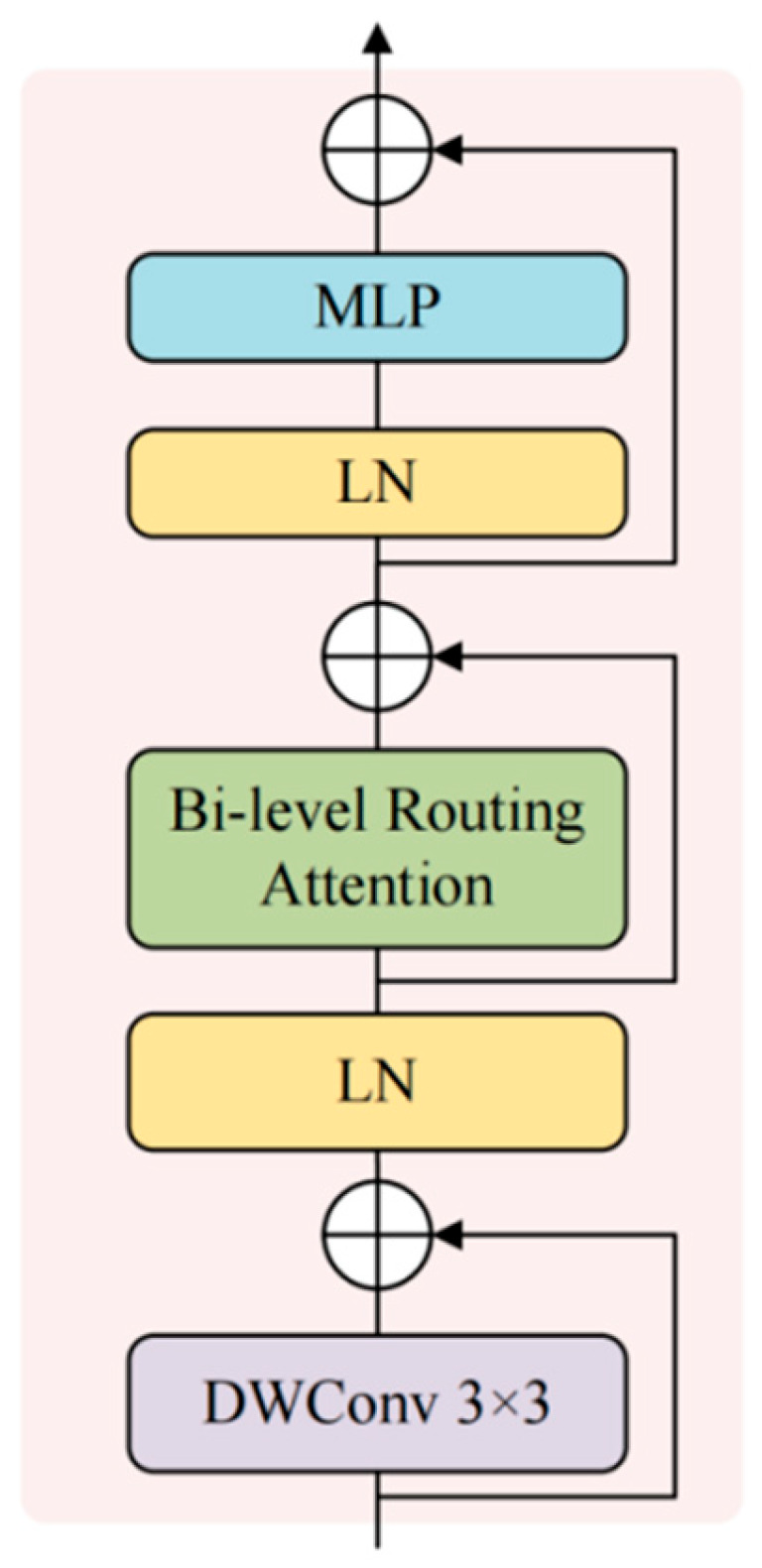
The resolution of both input and output of the GCRModule block after being inserted into ASPP remains H × W, consistent with the decoder interface.

The MLP consists of two linear layers with GELU activation and a default expansion ratio of 4. When necessary, a 1 × 1 linear layer is used to align the channel dimension C.  We set the grid size S=8, the number of routed candidates k=4, and the number of attention heads to 8, which complements the multi-scale context of ASPP without changing the resolution. Finally, $X_{gcr}$ is upsampled by a factor of 4 to $\frac{H}{4}\cdot \frac{W}{4}$ and fused with the shallow decoder features at the same scale, as shown in Figure 4.

Figure 4 shows the structure of the GCRModule Block. This block forms a local multi-scale interaction with the ASPP, serving as a global supplement before the decoder. In the routing stage, the S×S grid regions are formed based on the area similarity, and *top-*k related regions are selected. The feature map is further enhanced with a 3 × 3 depth convolution and MLP, ensuring stability and proper linear continuity. The final output, $X_{gcr}$, is then fused with the decoder’s shallow features at the same resolution.

### 2.4. DPFModule

**Figure 5:**
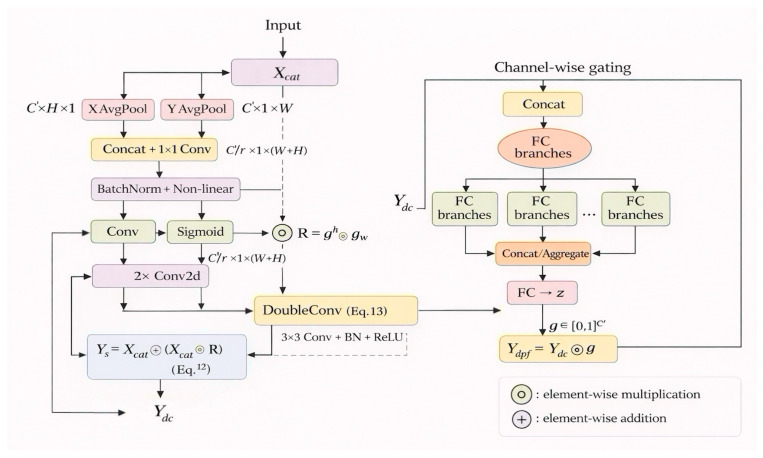
Cascade structure of the DPFModule.

The decoder combines the high-level features with low-level fine-grained features through concatenation at the same spatial resolution, followed by a 1 × 1 convolution for channel alignment, yielding $X_{cat}\in R^{\frac{H}{4}\cdot \frac{W}{4}\times C^{\prime }}$. At the same time, it enhances the global and local context sensitivity, as well as edge refinement and channel selection. After concatenation, we introduce the Directional Perception Fusion (DPF) module to perform direction-aware spatial re-weighting and channel-wise gating. It adjusts the attention direction to align with the local refinement and then passes through the sequential aggregation [25]. This design suppresses texture-induced scattered activations via direction-consistent spatial weighting R, and refines object boundaries by residual-preserved re-weighting followed by channel-wise gating. The spatially refined feature after Equation (13) is denoted as $Y_{dc}$. For compactness, we place $Y_{dc}$ at the bottom-left and also mark it as the input of the channel-wise gating branch; the explicit connecting arrow from $Y_{dc}$ to the gating branch is omitted. In Figure 5, ⊙ and ⊕ explicitly denote element-wise multiplication and addition, respectively, corresponding to the spatial re-weighting in Equation (12) and the final gating in Equation (17).

First, perform 1D global pooling along the row and column directions to obtain statistical values in both directions, as shown below:

(11) $$z_{c}^{h}(h)=\frac{1}{W}\sum_{i=0}^{W-1}X_{c}(h,i),z_{c}^{w}(w)=\frac{1}{H}\sum_{j=0}^{H-1}X_{c}(j,w)$$

Initially, we concatenate the pooled features along the spatial dimension and feed them into a shared 1 × 1 convolution to obtain an intermediate representation. Then, the representation is split into two branches to generate directional attention maps $g^{h}\in R^{C^{\prime }\times H\times 1}$ and $g^{w}\in R^{C^{\prime }\times 1\;\times W}$ for the vertical and horizontal directions, respectively. The spatial re-weighting map is computed by element-wise multiplication $R=g^{h}⊙g^{w}$, where ⊙ denotes element-wise multiplication with broadcasting. Following Figure 5, we apply spatial re-weighting with the following residual formulation:

(12) $$Y_{s}=X_{cat}⊕(X_{cat}⊙R),Y_{s}\in R^{\frac{H}{4}\times \frac{W}{4}\times C^{\prime }}$$

where ⊕ denotes element-wise addition. Because R is formed by the intersection of horizontal and vertical responses $\left(R=g^{h}⊙g^{w}\right)$, isolated noisy responses that are not direction-consistent are down-weighted, while elongated structures aligned with either direction are preserved. The residual addition ⊕ maintains the original feature support and stabilizes optimization, preventing over-suppression of true edges and thus improving boundary sharpness. Two layers of 3 × 3 convolution are added to $Y_{s}$, with each layer followed by normalization and ReLU activation. The residual branch is maintained, which helps to suppress the unwanted responses generated by enhanced edge features. This process is denoted as $Y_{dc}$, as expressed in:

(13) $$Y_{dc}=Conv3\times 3(BN\to ReLU(Conv3\times 3(BN\to ReLU(Y_{s}))))+Y_{s}$$

Subsequently, the concatenation is normalized without altering the spatial distribution, and multi-branch concatenation and re-normalization are performed for $Y_{dc}$. First, we apply global average pooling to obtain $s=GAP(Y_{dc})\in R^{C^{\prime }}$. In Figure 5, the GAP operator $s=GAP(Y_{dc})$ is omitted for simplicity; this is reflected by the direct connection from $Y_{dc}$ to the subsequent fully connected aggregation, shown as the FC→z path). Then, B branches are used, and each branch applies two layers of sensory detectors to perform a nonlinear transformation of s [26], which is defined for the b-th branch as:

(14) $$ub=W^{\left(b\right)}2\phi (W^{\left(b\right)}1s+b^{\left(b\right)}1)+b^{\left(b\right)}2$$

(15) $$W^{\left(b\right)}1\in R^{\frac{C^{\prime }}{r}\times C^{\prime }},W^{\left(b\right)}2\in R^{\frac{C^{\prime }}{r}\times \frac{C^{\prime }}{r}},b=1,\ldots ,B$$

Notably, r refers to the reduction in dimensions. Here, ϕ represents the ReLU function, which outputs the concatenated feature vector along the channel dimension $u=[u1;\ldots ;uB]\in R^{\frac{BC^{\prime }}{r}}$, followed by a linear transformation. The concatenated output is then mapped to the channel dimension and passed through the attention weights, as shown below:

(16) $$\begin{array}{c} z=W_{\mathrm{agg}}u+b_{\mathrm{agg}},g=\sigma (z)\in [0,1{]}^{C^{\prime }},W_{\mathrm{agg}}\in R^{C^{\prime }\times \frac{BC^{\prime }}{r}} \end{array}$$

Here, σ(.) denotes the sigmoid function that maps the channel response to [0, 1], producing a channel-wise gating $g\in [0,1{]}^{C^{\prime }}$. Finally, we apply the channel-wise gate to the refined feature via element-wise multiplication, where g is broadcast along the spatial dimensions to obtain the DPF output $Y_{dpf}$, as shown below:

(17) $$\begin{array}{c} Y_{dpf}=Y_{dc}⊙g \end{array}\;$$

The default setting for the number of branches and compression rate is both 4. This design allows for the simultaneous activation of multiple channels, making it more suitable for multi-class fine-grained representation. Additionally, the entire DPF branch maintains consistency with the decoder interface in both spatial scale and tensor shape.

### 2.5. Loss Function and Training Objective

The study conducts supervision on the LoveDA and ISPRS Potsdam datasets with a step length of 4, using a combination of cross-entropy (CE) and multi-class Dice loss to balance class accuracy and the recall of uneven regions. The output class logits from the classifier are denoted as $Z\in R^{H/4\times W/4\times K}$, and the class probabilities P=softmax(Z) are calculated using the softmax function, where $G\in \{0,\ldots ,K-1255{\}}^{\frac{H}{4}\times \frac{W}{4}}$ for the range of classes, and 255 represents invalid pixels. The mask M is defined as 1[G≠255], indicating the valid pixel area. The classification loss is computed by:

(18) $$L_{CE}=-\frac{1}{\left|M\right|}\underset{(i,j):M_{ij}=1}{\sum }log\;P_{i,j,G_{i,j}}$$

Let $Y_{k}=1[G=k]$ denote the one-hot label for the k-th class; we define the corresponding Dice coefficient and the macro-averaged multi-class Dice loss as follows:

(19) $${Dice}_{k}\frac{2\langle P_{k},Y_{k}⊙M\rangle +\varepsilon }{∥P_{k}{∥}^{2}+∥Y_{k}⊙M{∥}^{2}+\varepsilon },\;\;\;\;\;\varepsilon =10^{-5}$$

(20) $$L_{Dice}=1-\frac{1}{K}\sum_{k=1}^{K}{Dice}_{k}$$

The final objective is a weighted sum of cross-entropy and macro-averaged Dice losses that balances calibration and overlap quality, with $\lambda_{CE}=\lambda_{Dice}=2.0$; it is computed only on valid pixels, as follows:

(21) $$L=\lambda_{CE}L_{CE}+\lambda_{Dice}L_{Dice},\lambda_{CE}=\lambda_{Dice}=2.0$$

Cross-entropy focuses on the consistency of pixel-level class labels and classification accuracy. Dice focuses on balancing class distribution, fine-grained structures, and sparsity. Under the goal of improving regional consistency and recall, the above loss avoids position calculation when M = 1, avoiding optimization of ineffective areas in the data or its own sparse regions. An ε is used to stabilize the values. In cases where there is a complete class deficiency in certain classes in the dataset, the average per class can be selected to appear only in certain cases without affecting the conclusions.

### 2.6. Experimental Setup

The computational hardware used in this study consisted of a workstation running Ubuntu 24.04.3 LTS and four NVIDIA GeForce RTX 3090 GPUs, each with 24 GB of memory. The software environment comprised Python 3.8 (Conda), PyTorch 1.10.0, torchvision 0.11.1, and MMCV-Full 1.7.0 built for CUDA 11.3 and PyTorch 1.10. Additional libraries included TensorBoard 2.11.0, timm, segmentation-models-pytorch, OpenCV-Python, einops, scikit-image, PyWavelets, pytorch_wavelets, and pytorch_msssim. We trained the model for 50,000 iterations with a per-GPU batch size of 4, using Adam with beta1 = 0.9 and beta2 = 0.999. The initial learning rate was 1 × 10^−3^ and the weight decay was 5 × 10^−3^; the learning rate followed a polynomial decay schedule with power 0.9 and a minimum of 1 × 10^−5^. For LoveDA, images were resized to 1024 × 1024 and randomly cropped to 512 × 512; for ISPRS Potsdam, we used pre-sliced 512 × 512 patches. We applied only photometric augmentations—color and brightness—and standard intensity normalization, while geometric transforms were disabled to keep comparisons controlled. At inference, we used single-scale sliding-window evaluation with a 512 × 512 window and a 256 stride, averaging predictions over overlaps to obtain the final segmentation at the original image resolution. Pixels labeled 255 were treated as invalid and ignored during loss computation and metric evaluation. Key training parameters are summarized in Table 1.

## 3. Experiment

### 3.1. Dataset and Evaluation Protocol

#### 3.1.1. Loveda Dataset

**Figure 6:**
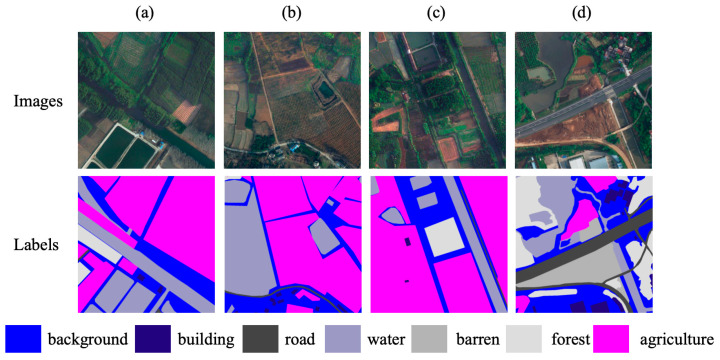
Examples from the LoveDA dataset. (**a**–**d**): RGB tiles with a size of 1024 × 1024 pixels from urban and rural areas. The bottom row shows the corresponding pixel-level annotations, where each color represents a distinct land-cover class. These annotations are used to assess the model’s ability to differentiate between various land types, including background, buildings, roads, water, barren areas, forests, and agriculture. The color legend is provided below for easy reference.

LoveDA is an urban–rural dual-domain semantic segmentation dataset with RGB tiles collected from cities including Nanjing, Changzhou, and Wuhan [27]. Each tile has a ground sampling distance of approximately 0.3 m and a size of 1024 × 1024 pixels. The imagery is acquired by high-resolution optical cameras on airborne or satellite platforms; in this work, we follow the common setting that uses only the three visible bands, treating the dataset as representative of camera-based land-cover mapping in operational remote sensing systems. The official split contains 2522 training images, 1669 validation images, and 1796 test images. The label set comprises seven categories: background, building, road, water, barren, forest, and agriculture. LoveDA spans dense urban scenes and sparsely populated rural environments with substantial scale variation and class imbalance, making it a widely used benchmark for assessing cross-domain robustness and boundary delineation. Figure 6 shows representative LoveDA RGB tiles and their pixel-level annotations for the seven classes. The preprocessing and evaluation protocols for LoveDA follow Section 2.3.

#### 3.1.2. ISPRS Potsdam Dataset

**Figure 7:**
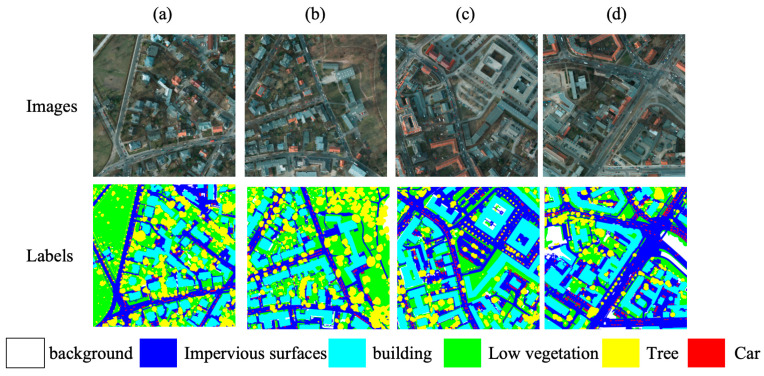
Examples from the ISPRS Potsdam dataset. (**a**–**d**): IRRG tiles with a ground sampling distance of 5 cm and a typical size of 6000 × 6000 pixels from urban areas. The bottom row shows the corresponding pixel-level annotations for the six classes. Each color corresponds to a different class, as indicated by the legend below the images.

The ISPRS Potsdam dataset contains orthorectified aerial images from urban areas. Each full tile has a typical size of 6000 × 6000 pixels and a ground sampling distance of 5 cm [28]. We use the IRRG three-channel imagery composed of near-infrared, red, and green bands. These tiles are captured by a very high-resolution digital aerial camera, and the inclusion of a near-infrared band makes the dataset representative of multi-band optical sensors widely used for urban monitoring. The dataset includes six categories: impervious surface, building, low vegetation, tree, car, and clutter/background. It covers common urban elements such as road networks, rooftops, and trees, which makes it suitable for assessing fine-grained segmentation and boundary delineation under high-resolution conditions. Figure 7 shows representative IRRG tiles and their pixel-level annotations for the six classes. For reproducibility, we adopt a fixed split with 30 full images for training and 7 for validation. The same seven images are used for testing. The 6000 × 6000 tiles are grid-sliced offline into 512 × 512 patches, and these patches are used directly for training. All remaining preprocessing and evaluation protocols follow Section 2.3.

#### 3.1.3. Evaluation Setup and Metrics

The evaluation is conducted using a single-scale sliding window, with the entire image predicted using a 512 × 512 window and a stride of 256. The category probabilities of the overlapping regions are averaged and fused to obtain segmentation results with the same dimensions as the original image. Unless otherwise specified, no multi-scale or mirroring augmentations are applied during testing. All metrics are calculated only on valid labeled pixels. For comprehensive comparisons, we adopt representative CNN-based baselines and recent Transformer-based baselines with publicly available implementations and consistent input modalities. All methods are trained and evaluated under the same protocol described above to ensure fair comparison.

Based on the definition of the metrics, for class c, the confusion counts are True Positive $TP_{c}$, False Positive $FP_{c}$, and False Negative $FN_{c}$. The IoU for class c is calculated using the following formula:

(22) $${IoU}_{c}=\frac{TP_{c}}{TP_{c}+FP_{c}+FN_{c}}$$

The precision $P_{c}$ and recall $R_{c}$ for class c are defined as $P_{c}=\frac{{TP}_{c}}{{TP}_{c}+{FP}_{c}}$ and $R_{c}=\frac{{TP}_{c}}{{TP}_{c}+{FN}_{c}}$, respectively. Accordingly, the F1 score for class c is defined as:

(23) $$F1_{c}=\frac{2P_{c}R_{c}}{P_{c}+R_{c}}=\frac{2TP_{c}}{2TP_{c}+FP_{c}+FN_{c}}$$

The pixel-level F1 score is equivalent to the Dice coefficient, so the values of mF1 and mDice in the following tables are the same. We report absolute gains in percentage points, denoted as pp. The macro-average mIoU and mF1 are given by:

(24) $$mIoU=\frac{1}{K}\sum_{c=1}^{K}IoU_{c},mF1=\frac{1}{K}\sum_{c=1}^{K}F1_{c}$$

Let M be the K × K pixel-level confusion matrix, then the overall accuracy (OA) is given by:

(25) $$OA=\frac{\sum_{c=1}^{K}{TP}_{c}}{\sum_{c=1}^{K}({TP}_{c}+{FN}_{c})}=\frac{tr(M)}{\sum_{i=1}^{K}\sum_{j=1}^{K}M_{ij}}$$

### 3.2. Comprehensive Comparison Experiments on the LoveDA Dataset

LoveDA covers both urban and rural domains and has an imbalanced class distribution, making it suitable for testing cross-domain robustness and multi-scale modeling capabilities. In a unified setup, we compare our method with representative CNN baselines and Transformer-based baselines, including BiSeNetV2 [29], PSPNet [30], ResUNet [31], SegFormer [32], and Swin-Tiny with UPerNet [33]. We evaluate the DeepLabV3+ baseline and our LGD-DeepLabV3+ under the same protocol, and the overall results are reported in Table 2.

Compared to the baseline, LGD-DeepLabV3+ achieves consistent improvements on LoveDA, with mIoU increasing from 49.65% to 58.48%, a gain of 8.83%; F1 improving from 65.48% to 73.37%, a gain of 7.89%; and OA rising from 71.39% to 76.32%, a gain of 4.93%. Compared to other methods, LGD-DeepLabV3+ achieves the best mIoU and F1 under the unified setting, indicating that LISModule, GCRModule, and DPFModule provide complementary gains in cross-domain scenarios. Class-wise comparisons are shown in Table 3.

**Figure 8:**
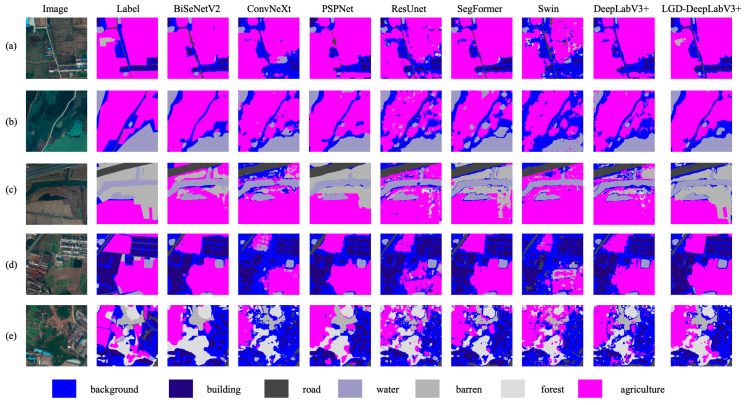
Qualitative comparisons on the LoveDA dataset. (**a**–**e**): Representative urban and rural tiles. Columns: Image, Label, BiSeNetV2, ConvNeXt + UPerNet, PSPNet (ResNet-18), ResUNet, SegFormer-B0, Swin-Tiny + UPerNet, DeepLabV3+ (ResNet-18), and LGD-DeepLabV3+ (ours). LGD-DeepLabV3+ demonstrates improved continuity of thin roads and rivers, clearer building boundaries under shadows, and fewer barren–water misclassifications compared to the baselines.

The improvement is particularly significant in slender or weak-texture categories. The IoU for barren increased by 17.87%, and F1 by 19.77%; the IoU for road increased by 11.28%, and F1 by 9.86%; the IoU for water increased by 9.95%, and F1 by 7.09%; agriculture and building also improved by 8.01% and 6.80%, respectively. Consistent qualitative effects are evident in Figure 8, where LGD-DeepLabV3+ yields more continuous thin roads and rivers, clearer building boundaries under shadows, and fewer barren–water confusions than the baselines.

### 3.3. Comprehensive Comparison Experiments on the ISPRS Potsdam Dataset

ISPRS Potsdam has a 5 cm resolution with rich small targets and clear boundaries, making it suitable for testing the overall capability of long-range modeling and boundary delineation. We follow the same evaluation protocol and comparison set as in Section 3.2 to ensure consistent and fair benchmarking, and the overall results are shown in Table 4.

Compared to the DeepLabV3+ baseline, LGD-DeepLabV3+ achieves comprehensive improvements on ISPRS Potsdam, with mIoU increasing from 74.07% to 80.79%, a gain of 6.72%; F1 improving from 84.60% to 89.16%, a gain of 4.56%; and OA rising from 86.72% to 90.34%, a gain of 3.62%. Compared to other methods, LGD–DeepLabV3+ achieves consistently strong mIoU and F1 under the unified setting, supporting the effectiveness of the proposed modules in high-resolution urban scenes. Class-wise comparisons are shown in Table 5.

**Figure 9:**
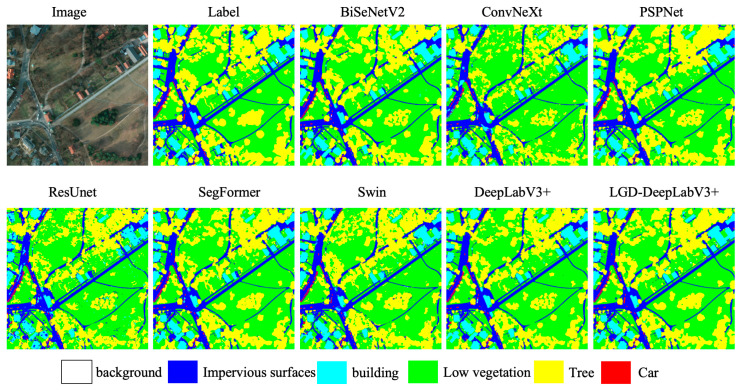
Qualitative comparisons on the Potsdam dataset. LGD-DeepLabV3+ demonstrates improved segmentation performance, particularly in boundary delineation and the continuity of thin structures, clearer building boundaries under shadows, and reduced barren–water misclassifications. The color legend below indicates the corresponding land-cover classes.

As seen in Table 5, all classes show improvements. Specifically, the IoU for background increased by 16.57%, and F1 by 12.59%; the IoU for impervious surfaces improved by 5.78%, and F1 by 3.36%; the IoU for low vegetation and tree both improved by 5.22%, with F1 increasing by 3.45% and 3.54%, respectively; car and building also achieved IoU increases of 4.03% and 3.52%, with F1 improving by 2.51% and 1.92%, respectively. This indicates that the LISModule on the input side helps reshape the distribution of weak-texture regions, the GCRModule after ASPP enhances long-range consistency across regions, and the DPFModule at the decoder side suppresses false textures and improves boundary quality in shallow–deep fusion. The synergy of these three modules brings stable gains. Qualitative results are shown in Figure 9.

### 3.4. Ablation Study and Complexity Trade-Off

Under the same data processing, training, and evaluation protocol, we use DeepLabV3+ as the baseline and evaluate the effect of adding each proposed module individually, in pairwise combinations, and with all three modules integrated in the order shown in Figure 3, Figure 4 and Figure 5. Specifically, +LISModule indicates the pre-shaping with Laplacian pyramid + KAN convolutions at the input side; +GCRModule refers to adding global context routing after ASPP; +DPFModule represents direction recalibration at the shallow-deep concatenation stage, followed by two 3 × 3 convolutions and channel aggregation; LGD-DeepLabV3+ represents the integration of all three modules in the order shown in Figure 3, Figure 4 and Figure 5. The remaining training and inference settings are consistent with the baseline. The evaluation uniformly reports OA, mIoU, and F1, along with the percentage improvement Δ relative to the baseline. Table 6 and Table 7 present the ablation comparisons on LoveDA and ISPRS Potsdam, respectively.

As shown in Table 6, each module yields a clear improvement over the baseline on LoveDA, while the gains from combining modules are not strictly additive and may show small fluctuations across different pairwise settings. After adding the LISModule, the improved input distribution leads to more stable recognition of weak-texture and complex background regions, with significant improvements in both mIoU and F1. After adding the GCRModule, long-range consistency is significantly enhanced without changing the resolution, with the most significant improvements seen in connected structures such as roads and rivers. After adding the DPFModule, boundaries and slender targets become clearer, with the largest improvement in F1. In LGD-DeepLabV3+, the complementary effects of the three modules result in the best performance, with mIoU increasing by +8.83 pp, F1 by +7.89 pp, and OA by +4.93 pp. We note that the improvements brought by the three modules are not expected to be strictly additive. This is because they partially address overlapping error patterns (e.g., confusing textured backgrounds and boundary ambiguities). Once a dominant source of errors is mitigated by one module, the remaining errors become harder, leading to diminishing marginal returns when stacking multiple modules. Nevertheless, integrating all three modules still achieves the best overall scores, indicating that the modules are complementary in a practical sense. Table 7 presents the ablation comparison on ISPRS Potsdam.

In Potsdam, where large man-made structures exhibit strong long-range regularities, GCR tends to contribute more to mIoU, while DPF consistently improves boundary-related F1, and LIS improves robustness in textured regions. LGD-DeepLabV3+ achieves the highest scores across all three metrics. The GCRModule primarily drives the improvement in mIoU, the LISModule provides stable robustness gains in complex textured backgrounds, and the DPFModule further refines boundaries and small targets. This indicates that in high-resolution urban scenarios, cross-region dependencies and global consistency are more critical. The synergy of LGD-DeepLabV3+ is evident, with mIoU improving by +6.72 pp, mF1 by +5.01 pp, and OA by +3.62 pp relative to the baseline.

The complexity and efficiency statistics are shown in Table 8. Here, the term ‘lightweight’ refers to the engineering/integration overhead (i.e., plug-and-play modules without redesigning the backbone/decoder interfaces), rather than claiming negligible absolute parameters or FLOPs. We, therefore, emphasize an accuracy–efficiency trade-off in the revised manuscript, all obtained under a unified inference setup. We report the baseline, the most computationally intensive single-module variant (GCR), and the full model, while LIS and DPF introduce comparatively minor overhead under the same inference setting. Latency and FPS are measured with the same input resolution, batch size, and hardware configuration, as described in the experimental setup. The baseline has the lightest computation and the lowest latency. When only the GCRModule is added, the parameter and memory overhead are moderate, providing stable improvements in mIoU and mF1 without changing the resolution or interface, making it suitable for latency-sensitive scenarios. After the three modules are concatenated to form LGD-DeepLabV3+, the computational and memory costs increase significantly, but the best and most balanced accuracy improvements across datasets are achieved, with notable improvements in long-range consistency, slender structures, and real boundary delineation. Overall, while the single modules provide low-cost, plug-and-play gains, the complete framework achieves the best accuracy within acceptable overhead.

## 4. Conclusions

This paper proposes LGD-DeepLabV3+ based on the DeepLabV3+ baseline, forming a collaborative framework with three pluggable enhancements that span input shaping, global modeling, and decoding refinement. The input-side LISModule reshapes the distribution and stabilizes optimization through Laplacian decomposition and lightweight KAN convolutions. The GCRModule, placed after ASPP, applies routing-based global modeling, focusing computation on relevant regions to strengthen long-range dependencies and cross-region consistency. The DPFModule at the decoder end enhances boundary and slender target delineation through direction recalibration, local refinement, and channel aggregation. Under a unified training and evaluation setup, the method achieves an 8.83% increase in mIoU and a 7.89% increase in mF1 on LoveDA, and a 6.72% increase in mIoU and a 4.56% increase in mF1 on ISPRS Potsdam, with OA also improving. Road and water network continuity is stronger, and building and shadow boundaries are clearer. Each module provides complementary gains with partial overlap, and the cascade achieves more balanced improvements in mIoU and mF1. The method can still maintain around 37.5 FPS during inference with a 512 × 512 input and batch size of 1, making it suitable for engineering integration and migration. The main bottlenecks currently lie in computational cost and cross-domain generalization. LIS is applied at the input level and is backbone-agnostic, so it can be used as a pre-processing front-end for CNN-, Transformer-, or Mamba-based segmentation models. GCR is inserted after ASPP in our implementation, but the routing-based global-context enhancement can also be attached to the high-level feature stage of Transformer decoders. DPF is designed for shallow–deep feature fusion in an encoder–decoder pipeline, and can be adapted to Transformer-based decoders that perform multi-scale fusion by applying the same directional recalibration and channel coupling on the fused feature maps. In this paper, we focus on improving the widely used DeepLabV3+ pipeline, and a full integration study on Transformer/Mamba backbones is left as future work. Future work will explore lightweight and adaptive solutions, including structural pruning to reduce redundancy [34], quantization to accelerate inference [35], and knowledge distillation to obtain compact student models [36], as well as dynamic routing, early stopping, domain adaptation, and multi-modal fusion, to further improve real-time performance and robustness in complex, large-scale scenarios. From a sensing perspective, LGD-DeepLabV3+ is agnostic to the specific camera or imaging sensor and operates directly on generic raster inputs. This makes it compatible with RGB/IRRG aerial cameras, multispectral satellite sensors, and multimodal image–DSM pairs similar to those used in recent sensor architectures such as LKAFFNet, MFPI-Net, RST-Net, and MMFNet. In future work, we plan to explicitly validate the framework on additional sensor modalities, including UAV video cameras, SAR–optical fusion, and LiDAR-derived height maps, further tightening the connection between advanced sensing hardware and semantic segmentation algorithms.

## Figures and Tables

**Table 1:** Model training parameters for LoveDA and ISPRS Potsdam.

Parameter	LoveDA	ISPRS Potsdam
Epochs	Iter-based (50,000 iterations)	Iter-based (50,000 iterations)
Batch size	4	4
Image size	Train crop: 512 × 512 Val/Test source: 1024 × 1024	Train crop: 512 × 512 Val/Test source: 6000 × 6000
Optimizer algorithm	Adam	Adam
Learning rate	0.001	0.001
Weight decay	0.005	0.005

**Table 2:** Comparison experiments on the LoveDA dataset.

Ex	Model	Model Variant	OA (%)	mIoU (%)	mF1 (%)
1	BiSeNetV2	-	73.58	54.36	69.91
2	ConvNeXt	Tiny + UPerNet	67.31	42.41	58.32
3	PSPNet	Resnet-18	71.17	50.72	66.68
4	ResUNet	-	68.03	46.57	62.35
5	SegFormer	B0	73.37	53.01	68.50
6	Swin	Tiny + UPerNet	63.95	39.14	55.23
7	DeepLabV3+	Resnet-18	71.39	49.65	65.48
8	LGD-DeepLabV3+	-	76.32	58.48	73.37

**Table 3:** LoveDA: Class-wise comparison between baseline and LGD-DeepLabV3+.

Class (K = 7)	Baseline mIoU (%)	LGD-DeepLabV3+ mIoU (%)	Baseline mF1 (%)	LGD-DeepLabV3+ mF1 (%)
background	57.36	60.17	72.91	75.13
building	52.42	59.22	68.78	74.39
road	45.68	56.96	62.72	72.58
water	62.57	72.52	76.98	84.07
barren	25.81	43.68	41.03	60.80
forest	44.17	49.14	61.28	65.90
agriculture	59.55	67.56	74.64	80.70

**Table 4:** Comparison experiments on the ISPRS Potsdam dataset.

Ex	Model	Model Variant	OA (%)	mIoU (%)	mF1 (%)
1	BiSeNetV2	-	88.16	76.16	86.16
2	ConvNeXt	Tiny + UPerNet	77.02	58.13	72.11
3	PSPNet	Resnet-18	84.91	69.96	81.85
4	ResUNet	-	78.14	59.70	72.54
5	SegFormer	B0	85.01	71.76	83.19
6	Swin	Tiny + UPerNet	79.67	63.53	76.88
7	DeepLabV3+	Resnet-18	86.72	74.07	84.60
8	LGD-DeepLabV3+	-	90.34	80.79	89.16

**Table 5:** ISPRS Potsdam: Class-wise comparison between baseline and LGD-DeepLabV3+.

Class (K = 6)	Baseline mIoU (%)	LGD-DeepLabV3+ mIoU (%)	Baseline mF1 (%)	LGD-DeepLabV3+ mF1 (%)
Background	54.16	70.73	70.26	82.85
Impervious surfaces	82.72	88.50	90.54	93.90
Building	89.99	93.51	94.73	96.65
Low vegetation	71.32	76.54	83.26	86.71
Tree	69.25	74.47	81.83	85.37
Car	76.99	81.02	87.00	89.51

**Table 6:** Ablation results on LoveDA, with values from the validation set; Δ denotes the absolute improvement over the baseline in percentage points (pp).

Ex	Method	OA (%)	mIoU (%)	mF1 (%)	ΔmIoU (pp)	ΔmF1 (pp)
1	DeepLabV3+	71.39	49.65	65.48	-	-
2	+LISModule	74.62	55.48	70.76	+5.83	+5.28
3	+GCRModule	74.73	56.07	71.32	+6.42	+5.84
4	+DPFModule	75.05	56.78	71.94	+7.13	+6.46
5	+LISModule+GCRModule	74.72	55.91	70.72	+6.26	+5.24
6	+LISModule+DPFModule	75.62	55.72	71.59	+6.07	+6.11
7	+GCRModule+DPFModule	75.93	57.54	72.76	+7.89	+7.28
8	LGD-DeepLabV3+	76.32	58.48	73.37	+8.83	+7.89

**Table 7:** Ablation results on ISPRS Potsdam, with values from the validation set.

Ex	Method	OA (%)	mIoU (%)	mF1 (%)	ΔmIoU (pp)	ΔmF1 (pp)
1	DeepLabV3+	86.72	74.07	84.60	-	-
2	+LISModule	88.75	77.54	86.98	+3.47	+2.38
3	+GCRModule	89.44	79.15	88.10	+5.08	+3.50
4	+DPFModule	89.39	78.97	87.97	+4.90	+3.37
5	+LISModule+GCRModule	88.73	79.65	87.65	+5.58	+3.05
6	+LISModule+DPFModule	89.43	79.52	88.97	+5.45	+4.37
7	+GCRModule+DPFModule	89.51	80.26	88.95	+6.19	+4.35
8	LGD-DeepLabV3+	90.34	80.79	89.16	+6.72	+4.56

**Table 8:** Comparison of complexity and efficiency.

Dataset	Method	Params (M)	GFLOPs (G)	Latency (ms)	FPS	PeakMem (GB)
LoveDA	Baseline	24.90	23.34	4.53	220.79	0.280
LoveDA	+GCRModule	103.66	43.50	6.15	162.51	0.575
LoveDA	LGD-DeepLabV3+	110.18	136.12	26.65	37.53	1.541
Potsdam	Baseline	24.89	23.33	4.50	222.12	0.277
Potsdam	+GCRModule	103.66	43.49	5.62	178.05	0.575
Potsdam	LGD-DeepLabV3+	110.18	136.11	26.70	37.46	1.541

## Data Availability

The original contributions presented in the research are included in the article; further inquiries can be directed to the corresponding author.
